# Influence of Family History of Diabetes on Health Care Provider Practice and Patient Behavior Among Nondiabetic Oregonians

**Published:** 2008-12-15

**Authors:** Amy I. Zlot, Mary Pat Bland, Kerry Silvey, Beth Epstein, Richard F. Leman, Beverly Mielke

**Affiliations:** Oregon Genetics Program, Public Health Division, Oregon Department of Human Services; Oregon Department of Human Services, Portland, Oregon; Oregon Department of Human Services, Portland, Oregon; Oregon Department of Human Services, Portland, Oregon; Oregon Department of Human Services, Portland, Oregon; Oregon Health and Science University, Portland, Oregon

## Abstract

**Introduction:**

People with a family history of diabetes are at increased risk of developing diabetes; however, the effect of family history of diabetes on health care provider practice and patient behavior has not been well defined.

**Methods:**

We analyzed data from the 2005 Oregon Behavioral Risk Factor Surveillance System, a state-based, random-digit–dialed telephone survey, to evaluate, among people with diabetes, associations between family history of diabetes and 1) patients' reports of health care provider practices, 2) patients' perceived risk of developing diabetes, and 3) patients' behaviors associated with an increased risk of developing diabetes.

**Results:**

Compared with respondents at average risk, respondents with a positive family history (strong or moderate familial risk for diabetes) were more likely to report that their health care provider collects family history information about diabetes, discusses the risk of developing diabetes or other chronic conditions, and makes recommendations to change their diet or exercise behaviors to reduce the chance of developing diabetes. Respondents with a strong family history of diabetes were 5 times more likely to be very or somewhat worried about developing diabetes than were people at average risk (odds ratio [OR], 5.0; 95% confidence interval [CI], 4.0-6.2). Compared with respondents at average risk, respondents with a strong family history were more likely to report making changes in diet and exercise (OR, 1.7; 95% CI, 1.4-2.1).

**Conclusion:**

Integrating family history of diabetes into clinical practice offers opportunities to improve the effectiveness of diabetes detection and to promote interventions aimed at preventing or delaying the development of diabetes in people at high risk.

## Introduction

Diabetes is a multifactorial disease that involves complex interactions between genes, environment, and health behavior. In 2006, the prevalence of clinically diagnosed diabetes reached almost 23 million people, or 7.5% of the US population (1). In 2005, the prevalence of self-reported diabetes among Oregonians was 6.7% of the adult population, an increase from 6.0% in 2003 and 4.0% in 1995 (1). At any given time, 30% of people with diabetes have not received a diagnosis (2).

Given the growing rate of diabetes and its far-reaching societal and economic consequences, prevention of diabetes among people at high risk is a public health issue of clinical importance. Identification of people at high risk is particularly important; strong evidence shows that the onset of type 2 diabetes can be reduced as much as 58% by moderate weight loss and healthy behaviors such as engaging in regular physical activity (3,4).

Genomics, which assesses the interaction of all of the genes in the genome with behavioral and environmental factors, offers potential for personalized prevention and treatment. Family history, which captures these genomic interactions, is considered clinically useful in the absence of genetic testing to identify people at high risk (5). A family history of diabetes is independently and significantly associated with the development of diabetes itself, even after adjusting for other risk factors (6). In general, a family history of diabetes in a first-degree relative doubles a person's risk of developing diabetes (5,7,8). People whose parents both have diabetes are 2 to 4 times as likely as people whose parents do not have the disease to develop diabetes themselves (9). The use of family history to identify people at increased risk for diabetes and perhaps motivate them to make preventive lifestyle changes could favorably affect both clinical practice and patient behavior.

The purpose of this study was to evaluate, among patients without diabetes, associations between family history of diabetes and patients' reports of health care provider practices, patients' perceived risk of developing diabetes, and patients' behaviors associated with diabetes.

## Methods

Data from the 2005 Oregon Behavioral Risk Factor Surveillance System (BRFSS), a state-based, random-digit−dialed telephone survey of health conditions and risk behaviors of the noninstitutionalized population aged 18 years or older, were used to estimate family history of diabetes, perception of diabetes risk, and behaviors associated with diabetes risk. Data were weighted by age, sex, and race/ethnicity to better reflect the demographic characteristics of Oregonians. More information about the Oregon BRFSS is available elsewhere (10). The Oregon Department of Human Services (DHS) deemed this project, which uses de-identified BRFSS data, to be exempt from institutional review.

### Survey measures

All respondents were asked, "Have you ever been told by a doctor that you have diabetes?" Respondents without diabetes were asked, "Do you have a parent, brother or sister, or child related by blood, who has been diagnosed with diabetes by a health care provider?" (Respondents were instructed not to include relatives who had diabetes only during pregnancy.) If they responded yes, they were asked to report the number of first-degree relatives with diabetes.

We classified respondents into average, moderate, or strong familial risk groups on the basis of the following factors (8): 1) average: no first-degree relatives with diabetes or adopted with unknown family history status of blood relatives; 2) moderate: 1 first-degree relative with diabetes; and 3) strong: at least 2 first-degree relatives with diabetes.

Respondents without diabetes were asked, "Has a doctor, nurse, or other health care provider ever discussed the chance of you getting diabetes?" "Has a health care provider ever recommended changes in diet or exercise to reduce your chances of getting diabetes or other illnesses like heart disease, stroke, or cancer?" "How worried are you that you will get diabetes in the future?" and "Have you made changes in your diet or exercise to reduce your chance of getting diabetes or other diseases like heart disease, stroke, or cancer?" Only respondents without diabetes who reported that their health care provider asked them, in general, about their family history of health problems were then asked, "Has a doctor, nurse, or other health care provider asked you about your family history of diabetes?"

### Covariates

Covariates, which may affect the association between family history and provider practice and patient behavior, include self-reported information on age, sex, education level, annual household income, race and ethnicity, physical activity level, obesity, diabetes, fruit and vegetable consumption, hypertension, cholesterol level, history of cholesterol screening, smoking status, insurance status, perceived health status, and having a personal doctor or health care provider. Respondents were considered to be of low socioeconomic status if household income was at or below 100% of federal poverty guidelines or if they had not completed high school. Respondents were categorized into the following physical activity levels on the basis of recommendations from the Centers for Disease Control and Prevention (CDC): meeting recommendations, insufficient activity, or inactive. CDC physical activity levels were defined as 1) recommended activity (either moderate-intensity activity during leisure time for 30 minutes or more on 5 or more days per week or vigorous-intensity physical activity during leisure time for 20 minutes or more on 3 or more days per week); 2) insufficient activity (some physical activity but not enough to meet CDC recommendations; or 3) inactive (less than 10 minutes of physical activity during leisure time in a usual week) (11). Obesity was defined as having a body mass index (BMI) greater than or equal to 30.0 kg/m^2^. Respondents were grouped by level of fruit and vegetable consumption as 1) consuming 5 or more servings of fruits and vegetables per day, or 2) consuming fewer than 5 servings a day. Respondents were asked if a doctor, nurse, or other health professional had ever told them that they had high blood pressure or high cholesterol. Respondents were also asked if they were trying to lose weight. Current smokers were respondents who reported smoking at least 100 cigarettes in their lifetime and currently smoking every day or some days.

### Data analysis

Pearson χ^2^ tests were used to detect differences in population attributes, patients' reports on provider practices, and selected behaviors among the 3 familial risk groups. Logistic regression was used to assess the association between family history and patients' reports of provider practices, perceived risk of diabetes, and selected behaviors. In the adjusted logistic regression models, only covariates that changed the point estimate of the odds ratio (OR) by at least 10% (compared with the full model) were kept in the final models. Interaction terms were included in the logistic regression models only if they were significantly associated with the outcome variables (*P* < .05).

All analyses were performed by using Stata version 9.2 (StataCorp LP, College Station, Texas), and the Taylor series linearization method was used to compute the variance of survey estimates in accordance with the complex sample design. Sample sizes (number of survey respondents) were reported as unweighted numbers.

## Results

The response rate for the Family History Section of the Oregon BRFSS was 52% or 6,688 survey respondents. Of the 6,688 respondents, we excluded 525 respondents who were told by a doctor that they had diabetes and 128 respondents with missing or unknown information regarding the number of family members with diabetes were excluded. A total of 6,039 respondents without diabetes were included in this analysis.

Among respondents without diabetes, approximately 27% had a family history of diabetes. Of these, 16.1% had 1 first-degree relative with diabetes (moderate familial risk), and 11.3% had 2 or more first-degree relatives with diabetes, indicating a strong familial risk. This translates into approximately 1 in 6 Oregonians without diabetes at moderate familial risk and 1 in 9 Oregonians without diabetes at strong familial risk for diabetes.

Younger people (aged 18 to 44 years) tended to be at average familial risk of diabetes compared with older people (aged 45 or older), which underscores the strong association of chronic diseases with increased age; a positive family history of chronic disease has a similar association. More women were at strong or moderate familial risk than were men. Latinos and people of a race other than white or Latino were more heavily represented in the strong risk category than were non-Latino whites. More obese people were at strong familial risk for diabetes (16.5%) than were nonobese people (9.5%) (Table 1).

More respondents who reported making lifestyle changes in their diet or exercise to reduce the chance of developing diabetes or other conditions were at strong or moderate familial risk of diabetes than were respondents who did not report making these lifestyle changes. Similarly, more respondents who reported that they were trying to lose weight were at strong or moderate familial risk compared with those who were not trying to lose weight. More respondents who reported having their cholesterol screened were at strong or moderate familial risk for developing diabetes than were respondents who did not have their cholesterol checked (Table 2).

Adjusted logistic regression analyses revealed that, compared with respondents at average risk of diabetes, respondents at strong or moderate familial risk were more likely to report that their health care provider collected information about family history of diabetes, discussed the risk of developing diabetes or other chronic conditions, and recommended lifestyle changes in diet or exercise to reduce the chance of developing diabetes or other chronic conditions. The magnitude of the OR was directly associated with familial risk (Table 3).

Respondents with a strong family history of diabetes were 5 times more likely to be very or somewhat worried about developing diabetes than respondents without a strong family history (OR, 5.0; 95% confidence interval [CI], 4.0-6.2). A family history of diabetes was associated with some protective behaviors but also with some conditions that place respondents at higher risk for developing the condition. Compared with respondents without a family history of diabetes, respondents with a strong or moderate family history of the disease were more likely to report making lifestyle changes in their diet or physical activity and more likely to report they were trying to lose weight. Respondents with a moderate family history were more likely to have their cholesterol screened than were respondents without a moderate family history (Table 3). However, respondents with a strong family history were twice as likely to be obese compared to people without a family history (OR, 1.9; 95% CI, 1.5-2.3). Family history was not a predictor of increased physical activity or fruit and vegetable consumption in these adjusted models.

## Discussion

Our study demonstrated that family history of diabetes is associated with a higher likelihood of patients' reporting that health care providers collect their family history information, discuss with them the risk of diabetes, and encourage them to make lifestyle changes to reduce the chance of developing diabetes. In addition, we found an association between family history and respondents' efforts to reduce their risk of developing diabetes. In contrast, a chart review of 516 patients without diabetes found no difference in primary health care providers' counseling rates for diet and physical activity or cholesterol testing based on patients' family history, although there was an association between familial risk and glucose testing (12). These differences in findings may be related to both provider documentation and patient-recall bias; our study was based on data from a self-reported population-based survey, whereas data in the other study were derived from medical records.

### Perceived risk and behavior change

Consistent with our results, other studies have found that people with a family history of diabetes  consider themselves to be at greater risk of developing diabetes than do people without a family history (13). Unfortunately, the link between increased risk perception and making behavior change is complex. Findings from other studies exploring the relationship between perceived risk of developing diabetes and behavior change are inconsistent (14). Some research suggests that people with a family history of diabetes perceive themselves to be at risk and engage in health-promoting behaviors, such as weight control and fruit and vegetable consumption, to decrease the risk of developing diabetes themselves (15). Conversely, another study found that increased perceived familial risk does not necessarily translate into motivation to change behavior, and some people may even adopt a fatalistic outlook (16).

Our study revealed that family history is associated with reported lifestyle changes that can reduce diabetes risk. People with a family history were more likely to report trying to lose weight than were people without a family history, although mean BMI scores did not vary according to family history status. people with a family history of diabetes were no more likely to meet CDC physical activity recommendations or to eat more fruits and vegetables than were people without a family history of diabetes. This finding could indicate that people with a family history of diabetes are changing their behavior in small increments but not enough to meet current physical activity or nutrition recommendations (17). Although we cannot confirm this theory from our cross-sectional BRFSS data, another possibility is that people with a family history of diabetes had worse baseline physical activity and nutrition levels than did those without a family history and that those with a family history have, in theory, over time made positive lifestyle changes but are not meeting national guidelines for these activities. In the same vein, people with a family history of diabetes may be making other behavior changes that we did not account for in our analyses. For example, people with a family history may be more likely to take a multivitamin or to try a fad diet than people without a family history, and neither of these behaviors was captured in our analyses. It also appeared that people with a family history are making positive behavior changes other than changes in diet and exercise. For example, in the Oregon BRFSS data, smokers with a family history of diabetes were more likely to report having stopped smoking for at least 1 day during the past year than were those without a family history (58.7% vs 50.4%, *P* = .03). In sum, the reasons that motivate particular people to change their behavior are multifaceted; family history is one of many factors, which under particular circumstances may influence people at high risk to engage in healthy, potentially protective behaviors.

The importance of respondents' stated readiness to make healthy lifestyle changes should not be dismissed. Successful weight management and a pattern of regular, sustained physical activity are often difficult to achieve. According to the Transtheoretical Model of behavior change, customizing interventions according to people's willingness to change can help health care providers use therapeutic resources more effectively and successfully (18,19). Numerous studies of structured interventions promoting moderate calorie restriction and regular physical activity have demonstrated the ability of people at high risk to achieve and sustain weight loss for up to 60 months (20). However, these interventions take time, willingness, and resources to be successful. Family history of diabetes, a potential motivating factor, may increase receptiveness to glucose screening and protective lifestyle changes among those who are predisposed to diabetes. Screening for glycemic control in this population could help identify people with prediabetes or undiagnosed diabetes who would be receptive to and could benefit from structured, evidence-based weight management and physical activity programs. Indeed, such screening is already recommended for persons with a family history of diabetes and a BMI >25 kg/m^2^ in the American Diabetes Association (ADA) Standards of Medical Care (21). Interventions that incorporate effective family history messages could motivate people at high risk to pursue and sustain healthy lifestyle changes. More research and resources targeting people who have a high familial and perceived risk but are not meeting lifestyle recommendations would be extremely useful in creating effective interventions for these high-risk populations, especially given our finding that people at high familial risk were almost twice as likely to be obese as were those in the average risk group.

Behavior change is influenced by a multitude of factors including perceived risk, self-efficacy (a person's assessment of ability to change behavior), social support (including support of health care providers), and environmental influences (eg, access to safe, pleasant areas for physical activity). The socioecological model asserts that effective interventions leading to healthy behaviors include a combination of efforts at all levels — individual, interpersonal, organizational, community, and public policy (22,23). To successfully incorporate awareness of family history into health promotion efforts, it must be combined with other tools that effectively initiate behavior change, and in turn reduce risks and improve individual health outcomes (16).

### Limitations

Several limitations should be considered when interpreting these findings. First, the stratification of familial risk of diabetes was loosely based on other established algorithms, but these algorithms have not been validated on a population-wide scale. Also, unlike the gold-standard algorithms for family history risk stratification, the risk categorization in our analysis did not account for second-degree relatives or size of the family (8,24). Although our analysis focused on Oregonians without diagnosed diabetes, the distribution of familial risk in our study sample was comparable to that in a recent national random survey, which also stratified the entire population by 3 risk levels for diabetes (6). We were unable to stratify the study sample by racial and ethnic groups besides non-Latino whites, Latinos, and other races because of small cell sizes. Causal associations cannot be drawn from this cross-sectional study, and because survey data were self-reported, they are subject to recall bias. However, self-reported information about diabetes, demographics, smoking status, and physical activity has been shown to be accurate, whereas BMI is likely to be underestimated (25). Also, having a family history of diabetes could influence how people perceive and remember their interactions with their health care providers. For example, people with a family history of diabetes may be more likely to recall that a provider collects family history information, discusses risk, and makes recommendations, whereas people without a family history may be more likely to forget these discussions. Lastly, this study does not distinguish between type 1 and type 2 diabetes, and family history and lifestyle factors have different effects on these conditions. However, this lack of distinction probably did not affect the results of our study because only 5% to 10% of all people with diabetes have type 1 (25).

### Risk stratification and targeting interventions

Our findings strengthen the evidence that collection and use of family history information is a useful genomics tool to prevent or delay the onset of diabetes in asymptomatic, at-risk populations among whom diabetes can be potentially prevented or delayed (7,8). In a recent study in Oregon, Kaiser Permanente clinicians reported that family history would be most useful to them if it were incorporated into algorithms and tools they already use to make clinical care decisions. Clinicians reported that they do not use family history information in isolation in their practices but that they adopt a holistic approach and use family history in conjunction with other factors to assess risk (26).

Incorporating family history information into diabetes risk assessment is a strategy that could help identify people who would benefit from diabetes prevention interventions and who are more likely to be receptive to such interventions. Most people can accurately report their family health history and believe it is important for their own health, which lends support to this strategy (27-30). Targeting asymptomatic people who are at risk for developing diabetes, on the basis of a combination of factors such as family history, BMI, and age, is likely to be an effective approach to limit the impact of diabetes on a population scale. Our findings warrant other studies, which could evaluate whether the ADA guidelines on screening for diabetes are actually being implemented in clinical settings. Such studies would serve as a crucial background piece for future provider and patient education and outreach efforts. Although more prospective studies are needed, various behavioral change theories support the hypothesis that people who are at highest risk are most likely to be receptive to adopting health interventions, such as healthy eating and physical activity behaviors (31).

## Figures and Tables

**Table 1:** Familial Risk of Diabetes Among Respondents Without Diabetes, 2005 Oregon Behavioral Risk Factor Surveillance System

Variable	**Total Sample N = 6,039^a^**	**Average Risk^b^ n (%)**	**Moderate Risk^c^ n (%)**	**Strong Risk^d^ n (%)**
**Age, y**				
18-44	1,152	887 (78.4)	147 (11.9)	118 (9.7)
45-64	2,238	1,529 (68.7)	419 (18.7)	290 (12.6)
≥65	2,645	1,868 (71.5)	462 (17.1)	315 (11.4)
**Sex**				
Male	2,352	1,789 (76.7)	352 (14.2)	211 (9.1)
Female	3,687	2,496 (68.6)	677 (17.9)	514 (13.5)
**Education**				
High school graduate or less	2,147	1,504 (72.3)	344 (14.6)	299 (13.1)
Some college	1,847	1,281 (71.6)	334 (17.2)	232 (11.2)
College graduate	2,036	1,494 (73.9)	350 (16.9)	192 (9.2)
**Annual household income, $**				
<25,000	1,535	1,070 (72.4)	239 (13.9)	226 (13.7)
25,000-49,999	1,898	1,333 (71.2)	343 (17.5)	222 (11.4)
≥50,000	1,933	1,389 (72.7)	346 (17.5)	198 (9.8)
**Socioeconomic status^e^**				
Low	870	626 (74.0)	144 (15.3)	100 (10.7)
Not low	4,543	3,212 (72.4)	792 (16.5)	539 (11.1)
**Race/ethnicity**				
Non-Latino white	5,301	3,774 (72.9)	916 (16.5)	611 (10.6)
Latino or Hispanic	151	111 (75.8)	21 (12.1)	19 (12.1)
Other	587	400 (69.6)	92 (14.7)	95 (15.8)
**Body mass index**				
BMI <30 kg/m^2^	4,476	3,271 (74.6)	748 (15.9)	457 (9.5)
BMI ≥30 kg/m^2^	1,306	850 (67.3)	229 (16.2)	227 (16.5)
**Hypertension^f^**				
Yes	1,559	1,067 (68.2)	285 (18.2)	207 (13.6)
No	4,464	3,210 (73.8)	740 (15.5)	514 (10.7)
**High cholesterol^g^**				
Yes	1,734	1,168 (68.4)	330 (18.1)	236 (13.5)
No	3,075	2,197 (72.3)	530 (17.1)	348 (10.6)

a Percentages are weighted. Numbers for some variables do not total 6,039 because of missing data.

b Average risk indicates respondent had no first-degree relatives with diabetes, had unknown family history status, or was adopted with unknown family history status of blood relatives.

c Moderate risk indicates respondent had 1 first-degree relative with diabetes.

d Strong risk indicates respondent had at least 2 first-degree relatives with diabetes.

e Respondents were considered low socioeconomic status if household income was at or below 100% of federal poverty guidelines or if respondents had not graduated from high school.

f Respondents reported that a doctor, nurse, or other health professional had told them that they had high blood pressure.

g Respondents reported that a doctor, nurse, or other health professional had told them that they had high cholesterol.

**Table 2:** Provider Practices, Patients' Perceived Risk, and Select Behaviors by Familial Risk of Diabetes Among Respondents Without Diabetes, 2005 Oregon Behavioral Risk Factor Surveillance System

Variable	Total Population **N = 6,039^a^**	**Average Risk^b^ n (%)**	**Moderate Risk^c^ n (%)**	**Strong Risk^d^ n (%)**
**Collection of family history of diabetes^e^**				
Yes	3,681	2,373 (66.1)	736 (19.1)	572 (14.8)
No	1,375	1,132 (82.1)	165 (12.6)	78 (5.3)
**Discussion of risk by a health care provider**				
Yes	1,805	957 (54.0)	429 (23.5)	419 (22.5)
No	4,157	3,271 (79.8)	589 (13.3)	297 (6.9)
**Recommendations to change behavior by a health care provider**				
Yes	2,281	1,455 (64.8)	467 (19.7)	359 (15.5)
No	3,718	2,801 (76.7)	556 (14.2)	361 (9.1)
**Perceived risk of diabetes**				
Very or somewhat worried	741	311 (44.0)	188 (25.6)	242 (30.4)
Not at all or slightly worried	5,262	3,956 (77.0)	834 (14.7)	472 (8.3)
**Reported lifestyle changes to reduce risk of diabetes**				
Yes	3,738	2,522 (68.4)	697 (18.2)	519 (13.4)
No	2,250	1,722 (78.5)	325 (13.1)	203 (8.4)
**Trying to lose weight**				
Yes	2,105	1,397 (68.0)	406 (18.7)	302 (13.3)
No	2,178	1,656 (77.5)	321 (13.6)	201 (8.9)
**Current smoker^f^**				
Yes	1,059	744 (71.3)	167 (15.3)	148 (13.4)
No	4,831	3,545 (72.8)	713 (16.3)	573 (10.9)
**Physical activity**				
Met recommendations	3,298	2,321 (73.1)	559 (16.2)	418 (10.7)
Insufficient activity	1,982	1,386 (71.0)	349 (17.2)	247 (11.8)
No activity	511	361 (75.1)	75 (11.6)	75 (13.3)
**Fruit and vegetable consumption**				
<5 servings per day	4,263	3,006 (71.8)	742 (16.7)	515 (11.5)
≥5 servings per day	1,769	1,272 (74.6)	287 (14.5)	210 (10.9)
**Cholesterol screening**				
Yes	4,839	3,383 (70.9)	864 (17.4)	592 (11.7)
No	1,077	805 (76.3)	151 (13.2)	121 (10.5)

a Percentages are weighted. Numbers for some variables do not total 6,039 because of missing data.

b Average risk indicates respondent had no first-degree relatives with diabetes, had unknown family history status, or was adopted with unknown family history status of blood relatives.

c Moderate risk indicates respondent had 1 first-degree relative with diabetes.

d Strong risk indicates respondent had at least 2 first-degree relatives with diabetes.

e Among respondents who reported that their health care provider collects family history information, in general.

f Current smokers were respondents who reported smoking who reported smoking at least 100 cigarettes in their lifetime and currently smoking every day or some days.

**Table 3:** Familial Risk of Diabetes as a Predictor of Provider Practice, Patients' Perceived Risk, and Select Behaviors Among Respondents Without Diabetes, 2005 Oregon Behavioral Risk Factor Surveillance System

**Dependent Variable**	Average Risk^a^ Adjusted OR(95% CI)	Moderate Risk^b^ Adjusted OR(95% CI)	Strong Risk^c^ Adjusted OR(95% CI)
Collection of family history of diabetes^d^	1.0 [Reference]	1.6 (1.3-2.0)	3.1 (2.3-4.2)
Discussion of risk of diabetes by a health care provider	1.0 [Reference]	2.0 (1.7-2.4)	3.9 (3.2-4.7)
Recommendations by a health care provider to make lifestyle changes	1.0 [Reference]	1.5 (1.3-1.8)	1.7 (1.4-2.0)^e^
Perceived risk of diabetes (very or somewhat worried vs not at all or slightly worried)	1.0 [Reference]	2.0 (1.6-2.5)	5.0 (4.0-6.2)^f^
Reported lifestyle changes to reduce risk of diabetes	1.0[Reference]	1.5 (1.2-1.7)	1.7 (1.4-2.1)
Current smoker^g^	1.0 [Reference]	0.9 (0.8-1.2)	1.2 (1.0-1.6)
Had cholesterol screening^h^	1.0 [Reference]	1.2 (1.1-1.7)	1.0 (0.8-1.4)
Physical activity (recommendations met vs recommendations not met)^i^	1.0 [Reference]	1.0 (0.8-1.2)	1.0 (0.8-1.2)
Fruit and vegetable consumption (≥5 servings per day vs <5 servings per day)^j^	1.0[Reference]	1.2 (1.0-1.4)	1.1 (0.9-1.3)
Obesity (BMI <30 kg/m^2^ vs BMI ≥30 kg/m^2^)	1.0 [Reference]	1.1 (0.8-1.2)	1.9 (1.5-2.3)
Trying to lose weight	1.0 [Reference]	1.4 (1.2-1.7)^k^	1.6 (1.3-2.0)

Abbreviations: OR, odds ratio; CI, confidence interval; BMI, body mass index.

a Average risk indicates respondent had no first-degree relatives with diabetes, had unknown family history status, or was adopted with unknown family history status of blood relatives.

b Moderate risk indicates respondent has 1 first-degree relative with diabetes.

c Strong risk indicates respondent has at least 2 first-degree relatives with diabetes.

d Among respondents who reported that their health care provider collects family history information, in general.

e Adjusted for obesity.

f Adjusted for age.

g Current smokers were respondents who reported smoking who reported smoking at least 100 cigarettes in their lifetime and currently smoking every day or some days.

h See Methods for complete definition.

i Adjusted for age, obesity, and smoking status.

j Adjusted for age and obesity.

k Adjusted for sex.
